# Within-City Average Life Expectancy “Gaps”: A Useful Health Equity Metric

**DOI:** 10.1007/s11524-025-01023-5

**Published:** 2026-01-26

**Authors:** Ben R. Spoer, Isabel S. Nelson, Matthew Lee, Anne Vierse, Alexander S. Chen, Andrea R. Titus, Lorna E. Thorpe, Marc N. Gourevitch

**Affiliations:** 1 Department of Population Health, Division of Epidemiology, New York University Grossman School of Medicine, New York, NY USA; 2 Department of Population Health, Division of Health and Behavior, New York University Grossman School of Medicine, New York, NY USA; 3 Independent Researcher, New York, NY USA; 4 Tulane University School of Medicine, New Orleans, LA USA

## Abstract

We characterize within-city life expectancy gaps and their correlation with social and environmental characteristics in 948 US cities. Life expectancy estimates were drawn from the US Life Expectancy Estimation Program. City life expectancy gaps were calculated by subtracting the lowest tract-level life expectancy estimate from the highest for each city. Correlations were established using Spearman’s correlation coefficient. The average city-level life expectancy gap in our sample was 11.8 years. Life expectancy gaps were larger in cities with lower average life expectancy and were evident across the USA. Life expectancy gaps of a decade were seen even in smaller cities and in high life expectancy cities. Life expectancy gaps were most strongly correlated with racialized residential segregation, children in poverty, and household income. Significant between-neighborhood gaps in life expectancy exist across US cities. Life expectancy gaps present a compelling target for establishing robust health equity goals.

## Introduction

Substantial geographic variation in average life expectancy (LE) persists across the USA. While public health research has focused more squarely on national-level drivers, less attention has been paid to modifiable drivers at the local level [1, 2].

Many jurisdictions use trends in LE to measure progress in improving resident health. Yet, while important, average LE can often mask important disparities, particularly in cities where conditions that affect LE can vary widely across small geographic areas. For example, LE is close to 87 years in New York City’s Upper East Side neighborhood, while four miles away in the South Bronx, LE is close to 75 years—a 12-year difference. Such knowledge can be galvanizing to efforts to prioritize intervention and investment in specific conditions that adversely influence health, with the goal of narrowing or eliminating a life expectancy gap. We hereafter refer to a within-city difference in LE as a “life expectancy gap” (LEG).

To date, there has not been a thorough description of city-level LEGs and their patterns across the USA. This research aims to describe LEGs as a robust health equity metric to inspire and guide local public health priority setting and interventions to improve health equity. A second aim is to investigate patterns of LEGs, overall and stratified by select social and environmental characteristics.

## Methods

### Study Sample

The city sample was drawn from the City Health Dashboard (www.cityhealthdashboard.com), a web-based resource that analyzes and presents data on health and its drivers for over 1200 US cities [3]. Cities missing tract-level LE data or with fewer than ten census tracts were removed to reduce unstable estimates, resulting in a sample of 948 cities. When stratifying by city-level LE, we additionally removed 28 cities that were missing tract LE data for more than 30% of the city population.

### Calculating Life Expectancy Gaps

Census tract-level LE estimates were obtained from the US Small-Area Life Expectancy Estimates Project (USALEEP). USALEEP modeled tract-level LE, accounting for small populations and missing death records and including various sociodemographic variables [4]. We used USALEEP LE modeled estimates from 2010 to 2015 (the most recent year of data available) to calculate LEGs by subtracting the lowest census tract-level LE value from the highest tract-level LE value for each city (Appendix Fig. 2). We additionally calculated LEGs using the fifth and 95th percentile tract LE values as a sensitivity analysis to address the potential for outlier tracts to influence the results.

### Selected City-Level Characteristics and Correlation Analysis

We visualized LEGs stratified by quartile of city-level LE. City-level LE was calculated using a block-level population weighting method to aggregate tract values to the city level, in consultation with USALEEP [3]. We additionally calculated average LEGs for cities across quartiles of 2015 population and calculated average LEGs for each census division to assess geographic patterns across the USA.

We selected ten city-level social and environmental characteristics by which to compare LEGs: median household income, percent of residents who completed high school or an equivalent degree, racialized residential segregation, count of tobacco stores per 1000 people, percent of the population lacking health insurance, percent of children in poverty, income inequality, percent of the population paying 30% or more of their income on rent, percent of the population living within a 10-min walk of a park, and walkability (Appendix Table 1). We used data from 2015, when available, to align with USALEEP. We calculated Spearman’s correlation coefficients between the numeric values of each city-level variable and city-level LEGs. We additionally presented average LEGs stratified by quartile of each city-level variable. These ten characteristics were selected to examine a range of structural factors that we anticipated would help contextualize LEGs within cities. As a sensitivity analysis, we also calculated correlations using LEGs defined by the fifth and 95th percentile tracts.

## Results

**Fig. 1:**
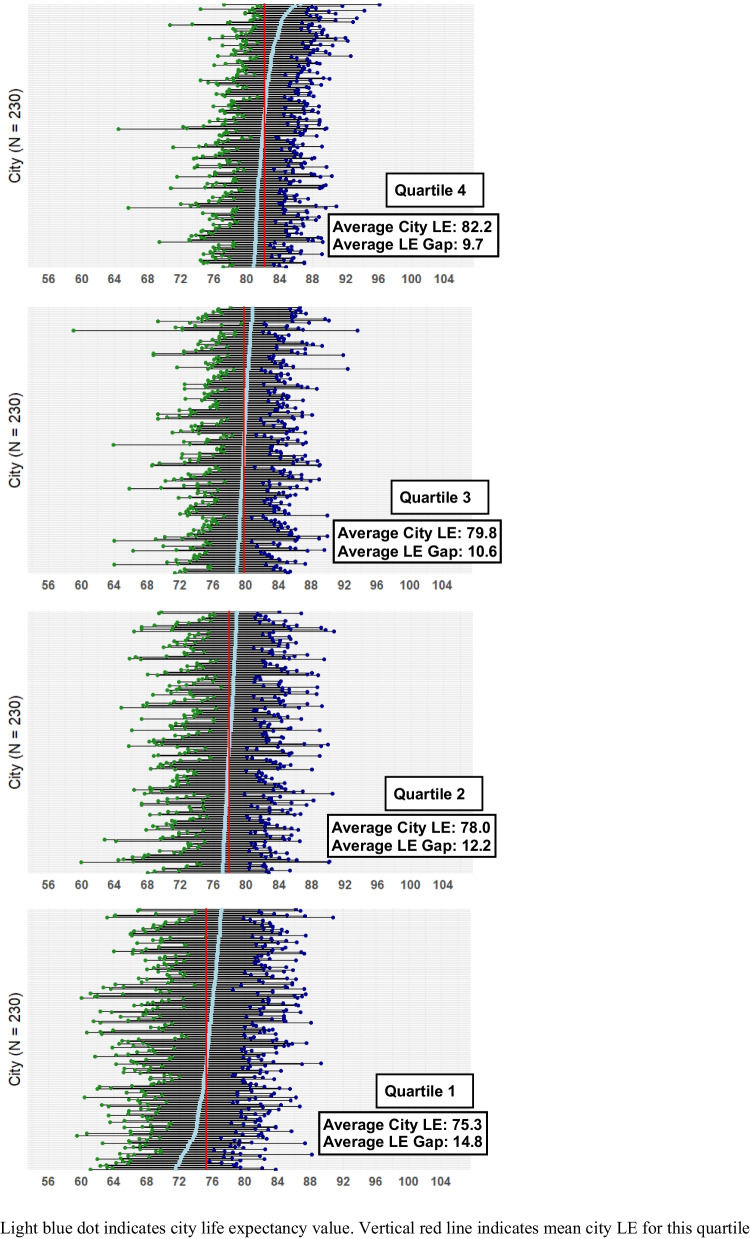
Life expectancy gap by quartile of city-level life expectancy

The average LEG across 948 cities in the study sample was 11.8 years. LEGs were larger in cities with lower LE. The average LEG in LE quartile one (average LE = 75.3 years) was 14.8 years, in quartile two (average LE = 78.0 years) 12.2 years, in quartile three (average LE = 79.8 years) 10.6 years, and in quartile four (average LE = 82.2 years) 9.7 years (Fig. 1). The figure depicts LEGs for all cities within each quartile of city-level LE. LEGs were larger in cities with larger populations; the average LEG in the smallest population quartile was 9.9 years and, in the largest quartile, 15.6 years (Appendix Table 2). Across census divisions, the average LEG ranged from 9.8 years in the New England division to 15.0 years in the East South-Central division (Appendix Table 2).

Among the social and environmental characteristics evaluated, city-level racialized residential segregation was most strongly and positively correlated with city-level LEGs (rho = 0.510). LEGs were negatively correlated (rho = − 0.447) with city-level median household income and positively correlated with city-level percent of children in poverty (rho = 0.458). Other factors had more moderate, weak, or no associations with city-level LEGs (Appendix Table 3). When average LEGs were stratified across quartiles of racialized residential segregation, percent children in poverty, and median household income, an approximately linear relationship was observed (Appendix Table 4). Correlation results were not substantially different when using the fifth and 95th percentiles to define LEGs (results not shown).

## Discussion

We examined LEGs as a potential measure of the “ground to cover” in setting city-level goals for health equity. LEGs were substantial across all geographic regions throughout the USA, with the highest average gap (15.0 years) observed in the East South-Central division. Even among smaller cities, where gaps are expected to be more modest due to fewer tracts and less potential for outliers, LEGs approximating a decade were commonly observed. Although LEGs decreased as city LE increased, we observed similar (decade-long) gaps in high-LE cities as well. These results are in line with prior research that has explored LEGs in a smaller number of major US [5, 6] and international cities [7] and research identifying strong associations between social drivers of health and similar mortality measures in urban geographies [8, 9]. The current study expands on this research by exploring these patterns in a larger number of US cities (including smaller-population cities) and by focusing on disparities within cities using granular spatial units rather than across demographic groups. The present research additionally suggests the potential of local urban LEGs as a useful tool for health equity goal setting.

Among social and environmental characteristics examined, the strongest correlation with LEGs was with racialized residential segregation, concordant with previous literature [10, 11]. While our descriptive analysis does not analyze potential mechanisms, it raises questions about the influences of environmental conditions, access to education and other resources, and the potential direct effects of exposure to racialized residential segregation on mental and physical health [12]. LEGs were also moderately correlated with city-level median household income and percentage of children in poverty. Substantial LEGs (averaging 9 years) were present even in higher-income cities, underscoring the need for local efforts to impact modifiable neighborhood characteristics to reduce disparities [13]. For example, initiatives to reduce food insecurity, support early childhood development, or optimize health insurance enrollment in specific neighborhoods may help to address within-city LEGs. A limitation of this analysis is that USALEEP estimates are from 2015, and LEGs may have since changed. Additionally, the income inequality variable used was calculated at the city level and so did not capture within-city variations in income inequality. This geographic mismatch between within-city LEGs and city-level income inequality may obscure potential correlation between income inequality and LEGs. Finally, particularly in larger cities, some census tracts may be outliers because of small or unique populations. Rather than asking cities whether specific outlier tracts should be excluded from analysis, as done in a preliminary analysis, our sensitivity and prior analyses suggest that outlier tracts do not skew our conclusions in this paper [14].

Several jurisdictions in the USA have begun to prioritize LEG reduction. The Cincinnati Department of Health is examining neighborhood-specific causes of premature death to inform interventions to reduce LEGs [15]. In Chicago, the Department of Health is using within-city LEGs to target strategies to reduce opioid overdose deaths [16]. The City of Boston published a report articulating similar goals in early 2025 [17, 18]. And New York City established a reduction in LEGs as a central aim of its effort to tailor health improvement strategies to specific neighborhood circumstances [19].

Our analyses demonstrate that significant LEGs exist across US cities. LEGs present a compelling target for municipal governments, health departments, healthcare systems, community organizations, and researchers to use in establishing robust health equity goals. Such efforts are beginning to take hold, and our analyses illuminate opportunities for cities to partner with communities to address neighborhood-level factors that impact LE disparities [20]. Future research can contribute to these efforts by exploring how specific drivers contribute to LEGs and by refining approaches to incorporate uncertainty in LE estimates. LEGs embody a powerful narrative that public health professionals and community leaders can and should leverage in advocating for and setting priorities to advance health equity.

## Figures and Tables

**Fig. 2:**
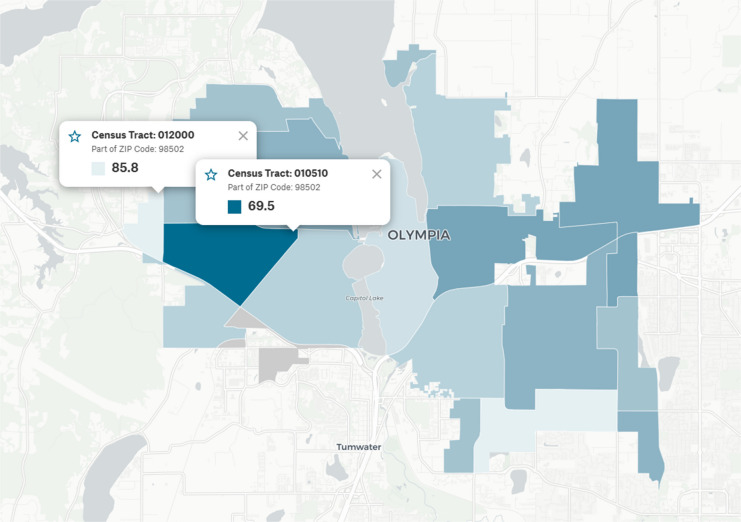
City-level example of a life expectancy gap in Olympia, WA. The pictured gap is 16.3 years

## Appendix

**Table 1:** Definitions and sources for additional variables

Variable	Definition	Source
Median household income	Median household income in dollars	ACS, 2015 5-year estimates, table S1901
% High school completion	Number of high school graduate, 25 years and older/total population, 25 years and older	ACS, 2015 5-year estimates, table S1501
Racialized residential segregation	Iceland’s entropy index using Theil’s *H*, multiplied by 100 to provide segregation scores ranging from 0 (no segregation) to 100 (absolute segregation)	ACS, 2015 5-year estimates, table DP05
Count of tobacco stores per 1000 people	Number of tobacco stores per 1000 people with sales > 0	National Neighborhood Data Archive (NaNDA): Liquor Tobacco, and Convenience Stores by Census Tract, US, 2003–2017
% Population lacking health insurance	Number of civilian noninstitutionalized population without health insurance, ages 0–64/number of civilian noninstitutionalized population, ages 0–64	ACS, 2015 5-year estimates, table S2701
% of children in poverty	Number of children aged < 18 living in households below the federal poverty level/total number of children aged < 18 living in households	ACS, 2015 5-year estimates, table B17020
Income inequality	(Count of households ≥ 80th percentile income − count of households ≤ 20th percentile income)/total number of households for which income is reported in the city. The absolute value of the final score is used for this paper, with lower numbers indicating lower inequality	ACS, 2015 5-year estimates, table B19001
% of population that is rent burdened	Households for which rent ≥ 30% of household income/total renter-occupied housing units with reported income	ACS, 2015 5-year estimates, table DP04
% of population living within a 10-min walk to a park	Population within a 10-min walk of green space/total population	ParkServe®, 2022
Walkability	Measure of the walkability of a location through incorporation of walking routes to nearby amenities from multiple categories (valued 0–100 with 100 being the most walkable). Population-weighted city-level average of census tract values	Walk Score, 2022
Total population	Total number of people living in the city	ACS, 2015 5-year estimates, table DP05

**Table 2:** Life expectancy gaps by total population and geographic location

	Mean LEG	Median LEG
Census division		
East South Central (AL, KY, MS, TN)	15.0	15.1
West North Central (IA, KS, MN, MO, NE, ND, SD)	13.4	12.2
South Atlantic (DE, DC, FL, GA, MD, NC, SC, VA, WV)	12.8	12.4
East North Central (IL, IN, MI, OH, WI)	12.7	12.4
West South Central (AR, LA, OK, TX)	12.2	12.1
Mountain (AZ, CO, ID, MT, NV, NM, UT, WY)	12.0	10.6
Middle Atlantic (NJ, NY, PA)	11.4	10.4
Pacific (AK, CA, HI, OR, WA)	10.2	9.8
New England (CT, ME, MA, NH, RI, VT)	9.8	9.5
City total population		
20,369–57,564	9.9	9.1
57,577–76,733	10.4	10.0
76,796–122,226	11.5	10.9
123,813–8,426,743	15.6	15.0

**Table 3:** Correlations between life expectancy gaps and selected city-level social and environmental characteristics

City-level metric	Correlation coefficient
Racialized residential segregation (*n* = 948)	0.510*
% of children in poverty (*n* = 946)	0.458*
Median household income (*n* = 946)	− 0.447*
% of population lacking health insurance (*n* = 946)	0.231*
% High school completion (*n* = 946)	− 0.188*
Income inequality (*n* = 873)	− 0.138*
% of population that is rent burdened (*n* = 946)	0.120*
Count of tobacco stores per 1000 people (*n* = 948)	0.089
% of population living within a 10-min walk to a park (*n* = 886)	− 0.021
Walkability (*n* = 948)	0.019

Sample size varies according to availability of covariate data

**p*-value < 0.001

**Table 4:** Life expectancy gaps stratified by city-level social and environmental characteristics

City-level metric by quartile	Mean LEG	Median LEG
Racialized residential segregation(*n* = 948)		
Quartile 1 (lowest)	9.2	8.9
Quartile 2	10.7	10.3
Quartile 3	11.9	11.4
Quartile 4 (highest)	15.4	14.7
% of children in poverty (*n* = 946)		
Quartile 1 (lowest)	9.0	8.5
Quartile 2	11.0	10.5
Quartile 3	13.0	12.2
Quartile 4 (highest)	14.4	13.7
Median household income (*n* = 946)		
Quartile 1 (lowest)	13.9	13.6
Quartile 2	13.2	12.2
Quartile 3	11.0	10.4
Quartile 4 (highest)	9.1	8.6
% of population lacking health insurance (*n* = 946)		
Quartile 1 (lowest)	10.2	9.3
Quartile 2	11.7	10.7
Quartile 3	13.2	12.2
Quartile 4 (highest)	12.2	11.9
% High school completion (*n* = 946)		
Quartile 1 (lowest)	11.9	11.3
Quartile 2	13.7	13.3
Quartile 3	11.6	10.7
Quartile 4 (highest)	10.1	9.6
Income inequality (*n* = 873)		
Quartile 1 (lowest)	11.0	10.3
Quartile 2	11.9	10.8
Quartile 3	12.3	11.6
Quartile 4 (highest)	12.5	12.5
% of population that is rent burdened (*n* = 946)		
Quartile 1 (lowest)	10.4	9.7
Quartile 2	12.6	11.7
Quartile 3	12.8	12.3
Quartile 4 (highest)	11.5	11.2
Count of tobacco stores per 1000 people (*n* = 948)		
Quartile 1 (lowest)	11.3	10.8
Quartile 2	11.8	10.8
Quartile 3	12.2	11.4
Quartile 4 (highest)	12.0	11.4
% of population living within a 10-min walk to a park (*n* = 886)		
Quartile 1 (lowest)	12.0	11.4
Quartile 2	11.9	11.3
Quartile 3	12.0	11.0
Quartile 4 (highest)	11.9	10.8
Walkability (*n* = 948)		
Quartile 1 (lowest)	11.0	10.5
Quartile 2	12.3	11.8
Quartile 3	12.4	11.4
Quartile 4 (highest)	11.6	10.1

Sample size varies according to availability of covariate data
